# Structural and immunological characterization of an epitope within the PAN motif of ectodomain I in *Babesia bovis* apical membrane antigen 1 for vaccine development

**DOI:** 10.7717/peerj.11765

**Published:** 2021-07-16

**Authors:** Amarin Rittipornlertrak, Boondarika Nambooppha, Anucha Muenthaisong, Veerasak Punyapornwithaya, Saruda Tiwananthagorn, Yang-Tsung Chung, Bumduuren Tuvshintulga, Thillaiampalam Sivakumar, Naoaki Yokoyama, Nattawooti Sthitmatee

**Affiliations:** 1 Graduate School of Veterinary Sciences, Chiang Mai University, Muang, Chiang Mai, Thailand; 2 Department of Food Animal Clinic, Faculty of Veterinary Medicine, Chiang Mai University, Muang, Chiang Mai, Thailand; 3 Department of Veterinary Bioscience and Veterinary Public Health, Faculty of Veterinary Medicine, Chiang Mai University, Muang, Chiang Mai, Thailand; 4 Department of Veterinary Medicine, College of Veterinary Medicine, National Chung Hsing University, Taichung, Taichung, Taiwan; 5 National Research Center for Protozoan Diseases, Obihiro University of Agriculture and Veterinary Medicine, Obihiro, Hokkaido, Japan

**Keywords:** Apical membrane antigen 1, Babesia bovis, Invasion-inhibition, Growth-inhibition, Synthetic peptide

## Abstract

**Background:**

Bovine babesiosis caused by *Babesia bovis (B. bovi*s) has had a significant effect on the mobility and mortality rates of the cattle industry worldwide. Live-attenuated vaccines are currently being used in many endemic countries, but their wide use has been limited for a number of reasons. Although recombinant vaccines have been proposed as an alternative to live vaccines, such vaccines are not commercially available to date. Apical membrane antigen-1 (AMA-1) is one of the leading candidates in the development of a vaccine against diseases caused by apicomplexan parasite species. In *Plasmodium falciparum* (*P. falciparum*) AMA-1 (PfAMA-1), several antibodies against epitopes in the plasminogen, apple, and nematode (PAN) motif of PfAMA-1 domain I significantly inhibited parasite growth. Therefore, the purpose of this study was to predict an epitope from the PAN motif of domain I in the *B. bovis* AMA-1 (BbAMA-1) using a combination of linear and conformational B-cell epitope prediction software. The selected epitope was then bioinformatically analyzed, synthesized as a peptide (sBbAMA-1), and then used to immunize a rabbit. Subsequently, *in vitro* growth- and the invasion-inhibitory effects of the rabbit antiserum were immunologically characterized.

**Results:**

Our results demonstrated that the predicted BbAMA-1 epitope was located on the surface-exposed α-helix of the PAN motif in domain I at the apex area between residues 181 and 230 with six polymorphic sites. Subsequently, sBbAMA-1 elicited antibodies capable of recognizing the native BbAMA-1 in immunoassays. Furthermore, anti-serum against sBbAMA-1 was immunologically evaluated for its growth- and invasion-inhibitory effects on *B. bovis* merozoites *in vitro*. Our results demonstrated that the rabbit anti-sBbAMA-1 serum at a dilution of 1:5 significantly inhibited (*p* < 0.05) the growth of *B. bovis* merozoites by approximately 50–70% on days 3 and 4 of cultivation, along with the invasion of merozoites by approximately 60% within 4 h of incubation when compared to the control groups.

**Conclusion:**

Our results indicate that the epitope predicted from the PAN motif of BbAMA-1 domain I is neutralization-sensitive and may serve as a target antigen for vaccine development against bovine babesiosis caused by *B. bovis*.

## Introduction

Bovine babesiosis caused by *Babesia bovis* (*B. bovis*), which is transmitted by the cattle tick (*Rhipicephalus microplus*), has had a significant effect on the cattle industry worldwide. Importantly, morbidity and mortality rates vary greatly. Degrees of mortality can reach 50–100% in untreated animals infected with this organism (Spickler, 2018). The asexual multiplication of *B. bovis* merozoites in bovine red blood cells (RBCs) is known to result in hemolytic anemia. In addition, the adherence of parasitized RBCs to the endothelial cells of the capillaries of the brain and lungs manifests as respiratory and nervous signs, which then can lead to a fatal form of babesiosis known as cerebral babesiosis (Bock et al., 2004). In general, the control of bovine babesiosis, including that which is caused by *B. bovis*, would need to be based upon effective tick control, chemotherapy, and vaccinations (Bock et al., 2004; Gohil et al., 2013). With a number of published reports focusing on the drug resistance of *B. bovis* and the development of acaricide resistance in ticks to *B. bovis* (Tuvshintulga et al., 2019), vaccination is considered to be an efficient method of control (Moreau et al., 2015).

Currently, live attenuated vaccines are being used to immunize cattle against *B. bovis* in a number of endemic countries (Bock et al., 2004). Nevertheless, the production process system can be time-consuming because the donor cattle must be prepared at least 2 months in advance before the production process can be initiated. In addition, blood-derived live vaccines are further required to test the safety and potency of the vaccine by cattle inoculation and clinical follow up before it can be released for commercial use. Moreover, the possibility of contamination with other blood-borne pathogens is a major risk that is associated with the use of blood-based vaccines. There has been evidence of bovine leucosis virus contamination being associated with the bovine babesiosis vaccine. Thus, vaccines need to undergo strict quality controls in order to avoid pathogen dissemination and to meet the relevant standards of conduct for good manufacturing practices. Even though vaccinations with live vaccines confer significant levels of protection, convincing evidence of *B. bovis* vaccine failures has been reported and confirmed during the period of 1985–1990. These failures have been associated with strain variations between the vaccine itself and breakthrough strains in immunized animals. As has been mentioned previously, the wide use of such live attenuated vaccines is limited in endemic regions (De Vos & Bock, 2000; Bock et al., 2004; De Waal & Combrink, 2006; Shkap et al., 2007). Therefore, the use of novel vaccines, such as recombinant vaccines or subunit vaccines, have been proposed to overcome some of these limitations (Suarez et al., 2019). The current approach relies on high-throughput technologies in the fields of genomic, proteomic, immunomic, and bioinformatic tools for the identification and design of new conserved candidate vaccine antigens (Kennedy & Poland, 2011; Lemaire, Barbosa & Rihet, 2012) in order to overcome certain limitations especially with regard to the genetic variations that cause the vaccine to breakdown. Antigens containing conserved epitopes would help to address issues of ethnicity when compared to the variable epitopes (Chong & Khan, 2019a; Chong & Khan, 2019b). Importantly, recombinant vaccines are products of genetic engineering, where a harmless agent is programed to produce antigens of harmful pathogens. Therefore, this approach has substantial advantages over traditional vaccines in terms of safety because they are made up of highly purified and well-defined components and lack the ability to replicate, which will allow researchers to avoid the use of unwanted materials that are capable of initiating a deleterious host response (Hudu, Shinkafi & Shuaibu, 2016). In the last decade, several *B. bovis* antigens have been studied as candidates for the development of recombinant vaccines. Among them, the apical membrane antigen 1 (AMA-1), which is a microneme protein (MIC) that had been extensively studied as a promising malaria vaccine candidate (Remarque et al., 2008), was identified as a neutralization-sensitive antigen in *B. bovis* (Gaffar et al., 2004). The exact functional role of AMA-1 is still unknown. However, previous investigations have suggested that AMA-1 plays an important role in the invasion of *Plasmodium falciparum* (*P. falciparum*) merozoites into RBCs (Triglia et al., 2000; Yap et al., 2014). AMA-1 forms moving junctions between the merozoite and the RBC, and thereby facilitates the invasion of merozoite into the RBCs (Srinivasan et al., 2011). Importantly, antibodies to *P. falciparum* AMA-1 (PfAMA-1) were able to detect native antigens in immunoassays and inhibit the growth and invasion of merozoites *in vitro* (Hodder, Crewther & Anders, 2001; Dutta et al., 2002; Malkin et al., 2005). In immunized monkeys, AMA-1-induced protection against *P. falciparum* blood stage infection was found to be superior to the protection provided by several other antigens (Stowers et al., 2002; Srinivasan et al., 2017). Remarkably, the results from human trials indicated that the AMA-1 formulated in different adjuvants was safe, well-tolerated, and immunogenic (Dicko et al., 2007; Thera et al., 2010; Sirima et al., 2017).

However, the use of AMA-1 in malaria vaccine formulations is constrained due to its high genetic diversity (Escalante et al., 2001; Drew et al., 2012). In contrast, *B. bovis* AMA-1 (BbAMA-1) is highly conserved in *B. bovis* (Ramos et al., 2012; Rittipornlertrak et al., 2017), and therefore is a target for diagnostic assays (Barreda et al., 2020). BbAMA-1 is composed of three domains of ectodomain (domain I–III) (Gaffar et al., 2004). Previous studies have demonstrated that the antibodies raised against a recombinant protein could effectively encode the conserved central region against synthesized peptides based on the epitopes present in domains II and III, while the N-terminal region of domain I significantly inhibited the *in vitro* growth and invasion of *B. bovis* merozoites (Gaffar et al., 2004; Salama et al., 2013). In *P. falciparum*, it was suggested that the plasminogen, apple, and nematode (PAN) domain is presumably known to mediate the adhesion of other proteins or carbohydrate receptors to AMA-1 (Pizarro et al., 2005). There are several known antibodies against the epitopes in the PAN motif of PfAMA-1 domain I that could significantly inhibit parasite growth (Coley et al., 2001, 2006; Harris et al., 2005; Maskus et al., 2016). Therefore, we have hypothesized that the epitopes in the PAN motif of the BbAMA-1 domain I are neutralization-sensitive. However, the epitopes in the PAN motif of BbAMA-1 have not yet been investigated for their vaccine potential.

To test this hypothesis, in the present study, we predicted an epitope in the PAN motif of the BbAMA-1 domain I, using linear and conformational B-cell epitope prediction softwares (Rittipornlertrak et al., 2017). The predicted epitope was synthesized and the resultant synthetic peptide (sBbAMA-1) was used to immunize a rabbit. Subsequently, the effect of the obtained antiserum on the growth and invasion of *B. bovis* merozoites was evaluated *in vitro*.

## Materials & methods

### *In vitro* cultivation of *B. bovis*

The Texas (T2Bo) strain of *B. bovis* was maintained with purified bovine RBCs in serum-free GIT culture medium (WAKO Pure Chemical Industrial, Ltd., Osaka, Japan) supplemented with antibiotic-antimycotic solution (Sigma–Aldrich, Tokyo, Japan). Parasites were continuously cultured in an atmosphere of 5% O_2_ and 5% CO_2_ at 37 °C. The culture medium was changed daily (Tuvshintulga et al., 2019).

### Prediction, analysis, and synthesis of a B-cell epitope

A BbAMA-1 amino acid sequence retrieved from the UniProtKB database (entry no. A7ASF6) was subjected to bioinformatic analysis to predict both linear and conformational B-cell epitopes within the PAN motif of the BbAMA-1 domain I (residue 147–305) as has been described by Rittipornlertrak et al. (2017). B-cell linear epitopes were predicted using a combination of immunoinformatic methods including the ABCpred Prediction Server (http://www.imtech.res.in/raghava/abcpred), the BepiPred 2.0 server (http://www.cbs.dtu.dk/services/BepiPred/ or http://tools.iedb.org/bcell/) and the LBtope server (http://crdd.osdd.net/raghava/lbtope/). In addition, conformational epitopes, mostly of the B-cell epitope form, were predicted using CBtope (http://www.imtech.res.in/raghava/cbtope/). The epitopes with a high prediction score that was above the threshold setting value located within the PAN motif were choosen from each server to generate a final epitope by combining the results of all immunoinformatic tools. Finally, the region that contained the greatest consensus of epitopes predicted by the four immunoinformatic tools was chosen as the final potential epitope region. The genetic diversity of the predicted epitope was visualized by aligning the corresponding amino acid sequences from various *B. bovis* isolates and other apicomplexan parasites using the MUSCLE algorithm (http://www.ebi.ac.uk/Tools/msa/muscle/) (Edgar, 2004). An analysis of the predicted secondary structures was conducted with the I-TASSER server (https://zhanglab.ccmb.med.umich.edu/I-TASSER/) (Yang & Zhang, 2015) and compared to sequences obtained from *Babesia* spp. and other apicomplexan parasites. Moreover, the predictive model of the complete BbAMA-1 structure was analyzed using the Phyre2 (http://www.sbg.bio.ic.ac.uk/phyre2/html/page.cgi?id=index) (Kelley et al., 2015) and I-TASSER servers. The crystal structure of *Babesia divergens* (*B. divergens*) AMA-1 (BdAMA-1) was used as a template to generate a theoretical model (Tonkin et al., 2013). To render the protein structure and localize the predicted epitope, the EzMol software Version 1.20 was applied (Reynolds, Islam & Sternberg, 2018). Finally, the selected epitope was synthesized as a peptide (sBbAMA-1) (GenScript, China).

### Animals

Two 3–6-month-old New Zealand White female rabbits (Mlac:NZW) were obtained from the National Laboratory Animal Center, Mahidol University, Nakhon Pathom, Thailand and kept individually in cages without bedding at a temperature of 20–24 °C at a humidity of 55 ± 10% with a 12/12-h light/dark cycle. The rabbits were housed at the laboratory building, Faculty of Veterinary Medicine, Chiang Mai University, Thailand and were maintained with *ad libitum* access to food and water. Due to the final blood collection of the rabbits, generalized anesthesia was administered by intravenous injection of pentobarbitone sodium (Nembutal^®^, Columbus, OH, USA, 20 mg/kg). Blood collection was done using a 1-inch long, 18-gauge needle to penetrate the jugular vein. Blood was taken until the volume reached 50 ml and the rabbits were then euthanized by intravenous injection of overdosage pentobarbitone sodium (Nembutal^®^, Columbus, OH, USA, 90 mg/kg).

### Production of rabbit polyclonal anti-sBbAMA-1 antibody

To obtain rabbit polyclonal anti-sBbAMA-1 antibody, one New Zealand white rabbit was intramuscularly immunized with 100 µg of sBbAMA-1 formulated with Montanide^™^ (Seppic, Paris, France; 1:1 v/v (one ml)) four times at 2-week intervals. To obtain a negative control serum, the other rabbit was intramuscularly immunized with 0.5 ml of Montanide adjuvant only. Adverse events including pain, swelling at the injection site, and behavioral changes were monitored throughout the course of the experiment. Every week, serum samples were collected from immunized rabbits in order to determine the antibody titer using an indirect enzyme-linked immunosorbent assay (ELISA). Briefly, each well in 96-well immuno plates (Nunc-Immuno Plate MaxiSorp, Intermed, Roskildes, Denmark) was coated with 10 ng of sBbAMA-1 in a coating buffer (0.05 M carbonate bicarbonate buffer, pH 9.6) and then incubated overnight at 4 °C. The plates were washed thrice with a washing buffer (Phosphate-buffered saline (PBS) containing 0.05% Tween-20) and then blocked with a blocking buffer (5% skim milk in PBS). Rabbit serum samples at a dilution of 1:100 in the blocking buffer was added to the wells and they were then incubated at 37 °C for 1 h. After being washed thrice with washing buffer, horseradish peroxidase (HRP)-conjugated goat anti-rabbit IgG antibody (KPL, Gaithersburg, MD, USA) was added at a dilution of 1:2,000 in the blocking buffer and the samples were then incubated at 37 °C for 1 h. After being washed thrice with washing buffer, 3,3′,5,5′-tetramethylbenzidine (TMB) substrate (SeraCare Life Sciences, Gaithersburg, MD, USA) was added and the samples were incubated at room temperature in dark. The reaction was stopped with 2 M H_2_SO_4_ after 15 min of incubation. Absorbance at 450 nm was measured using an Accu Reader Microplate reader M965 (Metertech, Taipei, Taiwan R.O.C.). Two weeks after the last immunization, a final blood sample was collected.

### Recognition of native BbAMA-1 by Western blot analysis

The *B. bovis* lysate was prepared by following the method described by Yokoyama et al. (2002) with minor modifications. Briefly, *B. bovis* infected RBCs obtained from *in vitro* cultures were treated with 0.83% NH_4_Cl solution for 10 min at 37 °C and then washed thrice with cold PBS. The pellets containing the parasites were suspended in one ml of a lysis buffer (50 mM Tris-HCl (pH 7.6), 0.1% Triton X-100, 150 mM NaCl, 20% glycerol, 1 mM EDTA, 1 mM phenylmethylsulfonyl fluoride (PMSF), 1 mM dithiothreitol (DTT), 10 μg/ml pepstatin A and 10 μg/ml leupeptin), incubated on ice for 20 min, and then centrifuged at 18,000×*g* for 30 min at 4 °C. The supernatant of the clarified lysate was dialyzed overnight using a dialysis buffer (50 mM Tris-HCl (pH 7.6), 150 mM NaCl, 20% glycerol, 1 mM EDTA, 1 mM PMSF and 1 mM DTT). The dialysate was then centrifuged at 18,000×*g* for 30 min at 4 °C. Subsequently, the supernatant was analyzed to identify native BbAMA-1 by Western blot analysis. The lysate was separated by 12% sodium dodecyl sulfate polyacrylamide gel electrophoresis (SDS-PAGE) gel. The proteins obtained from SDS-PAGE gel were electrically transferred onto a nitrocellulose membrane (Merck Millipore^™^, Merck KGaA^©^, Darmstadt, DEU). The blotting time was 60 min at a constant voltage of 20 V. The membrane was then incubated with a blocking buffer (5% skim milk in PBS) for 1 h at room temperature with gentle shaking. After being washed thrice with washing buffer (PBS containing 0.05% Tween-20), the membrane was incubated with rabbit polyclonal anti-sBbAMA-1 serum (1:50 dilution) at 4 °C overnight. The membrane was probed with HRP-conjugated goat anti-rabbit IgG antibody (1:4,000 dilution; KPL, Gaithersburg, MD, USA). The membrane was incubated with gentle shaking at room temperature for 1 h and then washed three times with washing buffer. Finally, the reactions were visualized using a solution containing 3,3′-diaminobenzidine (DAB; Invitrogen, Carlsbad, CA, USA) and hydrogen peroxide (H_2_O_2_; Merck, Germany).

### Immunofluorescence antibody test (IFAT)

The immunofluorescence antibody test was carried out as has been described in previous studies (Terkawi et al., 2011) with minor modifications. *B. bovis*-infected RBCs were coated on indirect IFAT slides (Matsunami Glass Ind., Ltd, Osaka, Japan), air-dried, and then fixed in cold absolute acetone at −20 °C for 5 min. Ten microliters of the rabbit anti-sBbAMA-1 serum diluted at 1:40 in the blocking buffer (5% fetal bovine serum (FBS) in PBS) was added as the first antibody on the fixed smears and then incubated for 1 h at 37 °C in a moist chamber. After being washed once with PBST, Alexa-Fluor^®^ 488 conjugated goat anti-rabbit IgG (Molecular Probes, Invitrogen, Carlsbad, CA, USA) was subsequently applied (1:200 dilution in the blocking buffer) as a secondary antibody and then incubated for 30 min at 37 °C. The slides were washed thrice with PBST, mounted in 50% glycerol-PBS, and then examined using a fluorescent microscope (E400 Eclipse, Nikon, Kawasaki, Japan).

### Preparation of free merozoites by cold treatment

The method described by Ishizaki et al. (2016) was used to isolate free merozoites liberated from infected RBCs. A five ml sample of an *in vitro* culture of *B. bovis* with 30% parasitemia was incubated on ice for 2 h. The culture was then resuspended in five ml of GIT medium. The suspension was slowly overlaid onto two ml of 30% (1.043 g/ml density) Percoll/PBS solution (GE Healthcare, Buckinghamshire, UK) at the bottom of a 15 ml centrifuge tube (Corning, Corning, NY, USA). The tube was centrifuged at 280×*g* for 5 min and then at 330×*g* for 20 min at 4 °C. The medium along with the free merozoite layers were transferred carefully to a new tube and then centrifuged at 1,500×*g* for 5 min at 4 °C. The pellets containing free merozoites were washed twice with 20 ml of GIT medium and then suspended in one ml of GIT medium. A concentration of purified merozoites was calculated with a disposable hemo-cytometer (AR Brown, Tokyo, Japan). The viability of the merozoites was determined after they were stained with 6-carboxyfluorescein diacetate (6-CFDA; Invitrogen Corp., Carlsbad, CA, USA) and propidium iodide (PI; Dojindo, Kumamoto, Japan).

### *In vitro* growth-inhibition assay

Growth-inhibition assay was performed in 96-well culture plates (Nunc, Roskilde, Denmark) as described by Salama et al. (2013). One hundred and eighty microliters of GIT medium, GIT medium containing anti-sBbAMA-1 serum at 1:5, 1:10 and 1:20, or rabbit anti-Montanide adjuvant serum (negative control) at a 1:5 dilution was added into each well in triplicate. Then, 20 µl of bovine RBCs with 1% parasitemia was added into each of the wells. The plates were incubated for 4 days. The culture medium was replaced everyday with fresh medium containing an indicated dilution of antiserum. Giemsa-stained thin blood smears were prepared every 24 h and parasitemia was monitored by counting the infected RBCs among approximately 1,000 total RBCs using a light microscope. The percentage of growth inhibition was calculated as the rate of parasitemia reduction in the antibody-treated cultures when compared to the GIT-control. The assay was repeated twice.

### *In vitro* invasion-inhibition assay

The RBC invasion-inhibition assay was performed in triplicate in 96-well plates (Nunc, Roskilde, Denmark, Europe) according to the method employed by Ishizaki et al. (2016) with some modifications. Briefly, purified free merozoites (obtained from *B. bovis in vitro* culture) together with uninfected bovine RBCs with a multiplicity of infection (MOI) of 4.2 were added to the GIT medium containing rabbit anti-sBbAMA-1 serum at dilutions of 1:5 and 1:10. Rabbit anti-Montanide adjuvant serum at a dilution of 1:5 and GIT medium were used as the control. The culture plate was incubated and Giemsa-stained thin blood smears were prepared at 1, 2 and 4 h post-incubation. The percentage of parasitemia was evaluated under a light microscope based on approximately 3,000 observed RBCs. The percentage of invasion-inhibition was calculated based on the parasitemia reduction in the antibody-treated cultures when compared to the GIT-control. Experiments were carried out in two separate trials.

### Statistical analyses

Growth- and invasion-inhibition rates were analyzed by independent-samples Student’s *t* test. *P* values of <0.05 were considered statistically significant when compared to the control groups.

## Results

### Potential epitope of BbAMA-1 predicted in this study

**Figure 1:**
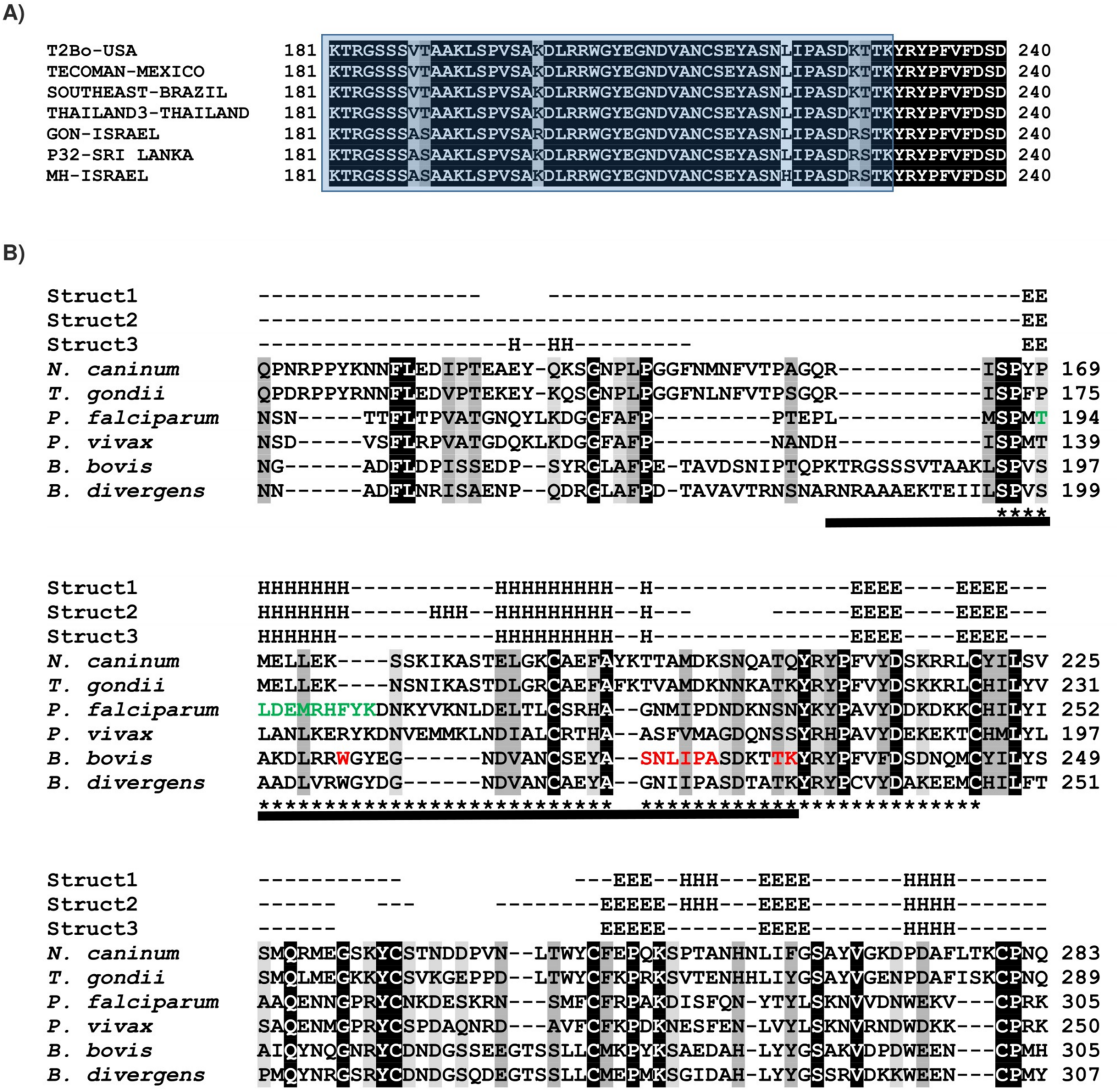
Sequence alignment of the BbAMA-1 with those sequences available in data base. (A) An alignment showing polymorphisms in the epitope predicted in the PAN motif of BbAMA-1 domain I (181–230) using immunoinformatics tools. The following sequences from GenBank were used to generate the alignment; XP_001611043, ASR91672, ACM44020, BAN78782, ANW86202, ANW86201 and QDK56771. The epitope region is highlighted in blue. (B) Alignment of the apicomplexan AMA-1 sequences correspond to PAN motif of PfAMA-1 domain I (Bai et al., 2005) from various apicomplexan parasite species. The GenBank-derived AMA-1 sequences of *Plasmodium vivax* (Q9TY14), *P*. *falciparum* (Q7KQK5), *Neospora caninum* (B6KAM0), *Toxoplasma gondii* (A2A114), *Babesia divergens* (C0IR59) and *B*. *bovis* (A7ASF6) were used in the alignment. The aligned secondary structure of AMA-1 from *P*. *falciparum* (Struct1, PDB: 4r1b), *P*. *vivax* (struct2, PDB: 1w8k) and *B*. *divergens* (Struct3, PDB: 4apm) are indicated as “E” for β strand and “H” for α helix. The epitope predicted in this study is indicated by a black bar. The epitope mapped by MAb 1F9 is indicated by asterisks (*). The MHC-restricted CD8+ T cell epitope in *P*. *falciparum* is highlighted with green letters. The residues associated with the invasion-inhibitory peptide R1 predicted by I-TASSER are highlighted with red letters.

**Figure 2:**
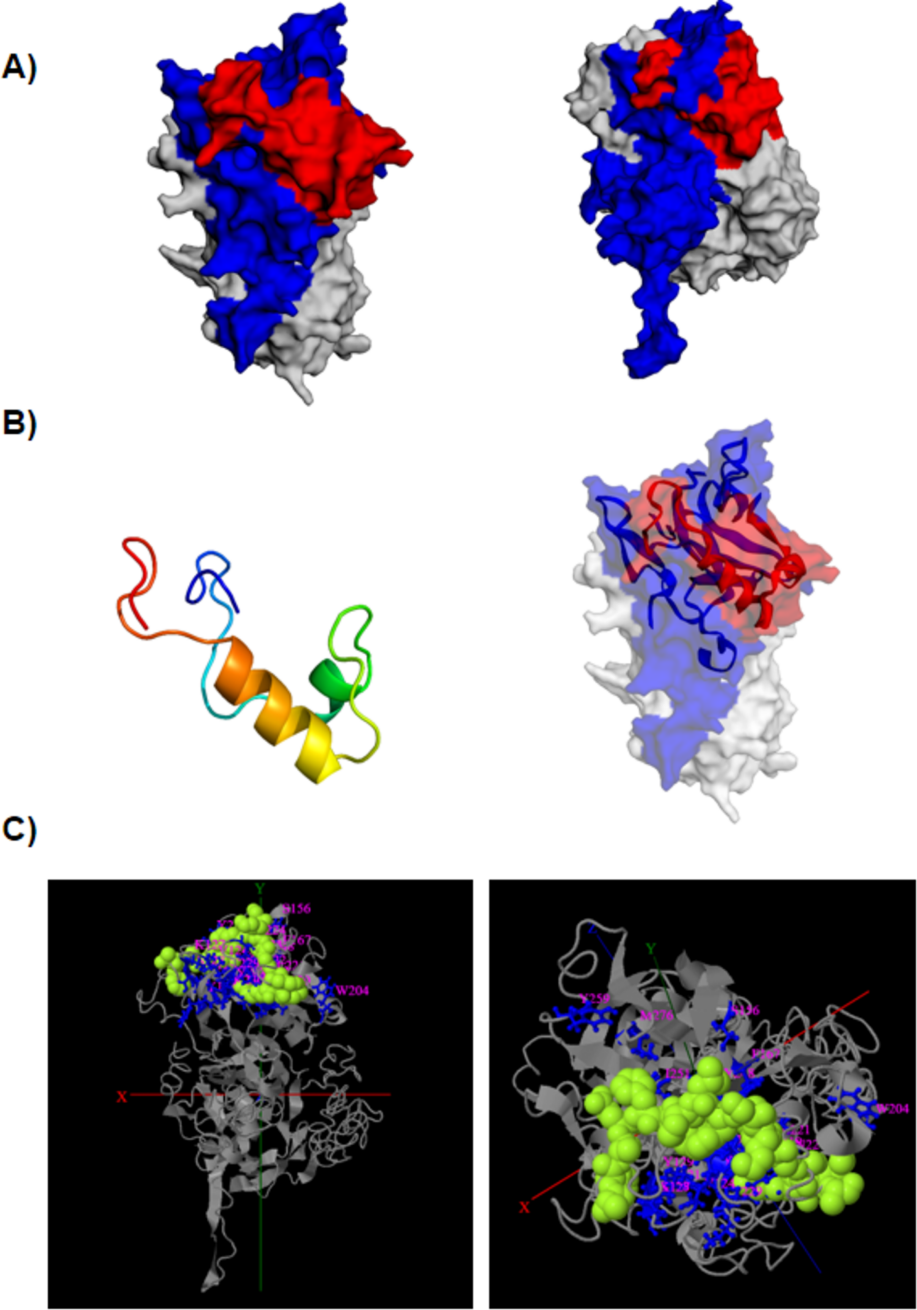
Homology modelling of BbAMA-1. (A) A homology model of the complete BbAMA-1 was constructed based on BdAMA-1 using Phyre2 and I-TASSER (Left). The final model of BbAMA-1 was construct by I-TASSER (Right). The structure showed the domain I of BbAMA-1 are represented by blue which contains the epitope areas predicted in this study indicated by red. The domain II-III are represented by grey. (B) The secondary structure of predicted epitope of BbAMA-1 form a-helix (Left) incorporated into the PAN motif of BbAMA-1 domain I represented by red based on BdAMA-1 structure (Right). (C) The invasion-inhibitory peptide R1 (PDB: 3srj, green atoms) associated predicted epitope residues (blue) analyzed by I-TASSER. The grey color structure is the BbAMA-1 constructed by I-TASSER.

Based on the BbAMA-1 sequence obtained from the Texas strain of *B. bovis*, an epitope, KTRGSSSVTAAKLSPVSAKDLRRWGYEGNDVANCSEYASNLIPASDKTTK, located between residues 181 and 230 was predicted within the PAN motif of ectodomain I using a combination of immunoinformatic tools. Multiple alignment of AMA-1 sequences obtained from different *B. bovis* isolates identified 6 polymorphic sites at residues 188, 189, 199, 221, 227 and 228 (Fig. 1A). Homology modelling indicated that BbAMA-1 is most structurally similar to *B. divergens* AMA-1 (BdAMA-1) (PDB: 4apm) with 65% identity, 56% coverage, and 0.558 of TM-score. Analysis of the BbAMA-1 protein structure revealed that the epitope residues were localized at the apex region of BbAMA-1 (Fig. 2A). According to the 3D model and predicted secondary structure of BbAMA-1, this epitope forms a surface-exposed α-helix of the PAN motif of the BbAMA-1 domain I (Fig. 2B). In *P. falciparum*, the invasion-inhibitory peptide R1 could inhibit the interaction between AMA-1 (PfAMA1) and rhoptry neck proteins (RON) (PfRON2), and thereby inhibit merozoite invasion (Harris et al., 2005). Interestingly, I-TASSER modelling indicated that the ligand-binding site of the invasion-inhibitory peptide R1 (PDB: 3srjB) is associated with the predicted epitope on BbAMA-1 (Fig. 2C). The possible binding sites for invasion-inhibitory peptide R1 in our epitope are the residues 204, 219, 220, 221, 222, 223, 224, 229 and 230 as are shown in Fig. 1B.

### Recognition of native protein by anti-sBbAMA-1 antibody

**Figure 3:**
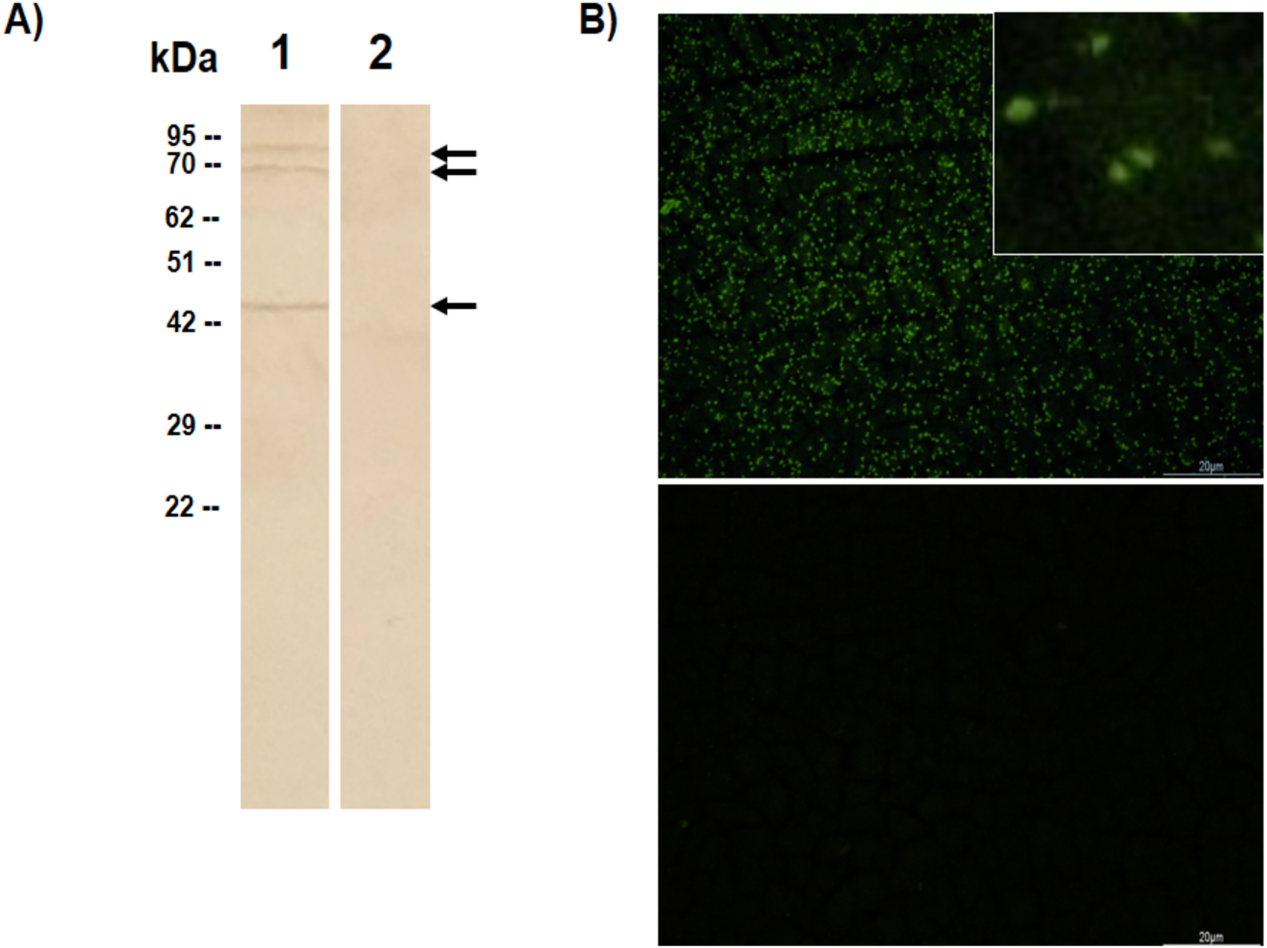
Recognition of native BbAMA-1 protein by rabbit polyclonal anti-sBbAMA-1 antibody. (A) Western blot assay was conducted to assess the reactivity of anti-sBbAMA-1 antibody with the native BbAMA-1. Lane 1: *B*. *bovis*-infected RBC lysate; lane 2: non-infected RBC lysate. (B) The immunofluorescence antibody test was performed to confirm the binding of sBbAMA-1 to the surface of *B*. *bovis* merozoites. The rabbit polyclonal anti-sBbAMA-1 serum (upper panel) or rabbit negative serum (lower panel) was incubated with *B*. *bovis*-infected RBSs at 1:5 dilution. Inset in upper panel show enlargement of *B. bovis* merozoites reacting with anti- sBbAMA-1. Scale bars = 20 μm.

In the Western blot analysis, the rabbit antiserum against sBbAMA-1 reacted with native proteins at sizes of approximately 82-, 69- and 45-kDa in the parasite lysate but not in the normal RBCs lysates (Fig. 3A). Furthermore, the anti-sBbAMA-1 antibody reacted strongly with *B. bovis* merozoites in IFAT, while no reactivity was observed when the negative control serum was used (Fig. 3B). These results indicate that the anti-sBbAMA-1 antibody was able to recognize native BbAMA-1 confirming the antigenicity of the predicted B-cell epitope.

### *In vitro* growth-inhibitory effect and morphology changes

**Figure 4:**
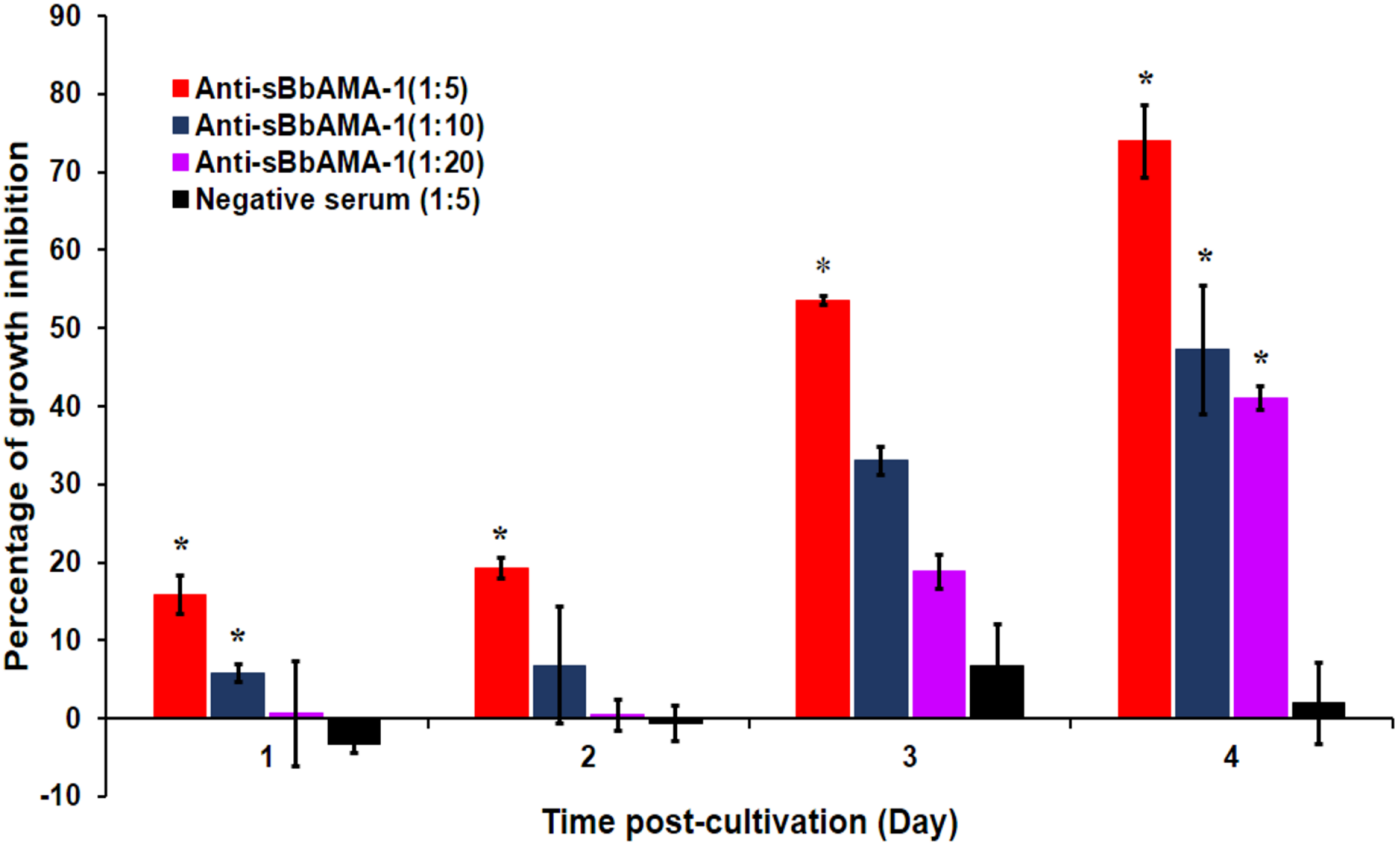
The growth-inhibitory effect of rabbit anti -sBbAMA-1 antibody on *B. bovis* merozoites. The *in vitro* growth inhibition assay was conducted with different dilution of anti-sBbAMA-1 serum and rabbit negative serum. The percentage of growth inhibition was calculated based on the parasitemia reduction in treated cultures compared to GIT-control. Asterisk indicates a significant difference (*p* < 0.05) in the percentage of growth inhibition in anti-sBbAMA-1 treated cultures compared to negative serum control.

To investigate the growth inhibitory effect of anti-sBbAMA-1 antibody, *B. bovis* was cultivated in media containing rabbit anti-sBbAMA-1 serum at dilutions of 1:5, 1:10 and 1:20. Subsequently, the parasitemia was monitored daily. The findings indicate that the anti-sBbAMA-1 antibody inhibited the parasite growth in a dilution-dependent manner, *i.e*., a dilution of 1:5 exhibited a higher inhibitory effect than the dilutions of 1:10 and 1:20, respectively (Fig. 4). The rates of growth inhibition induced by anti-sBbAMA-1 serum at a dilution of 1:5 were >50% and >70% on days 3 and 4 post-cultivation, respectively. In addition, parasite growth was significantly impeded at 1:10 and 1:20 anti-sBbAMA-1 dilutions on day 4 of cultivation.

**Figure 5:**
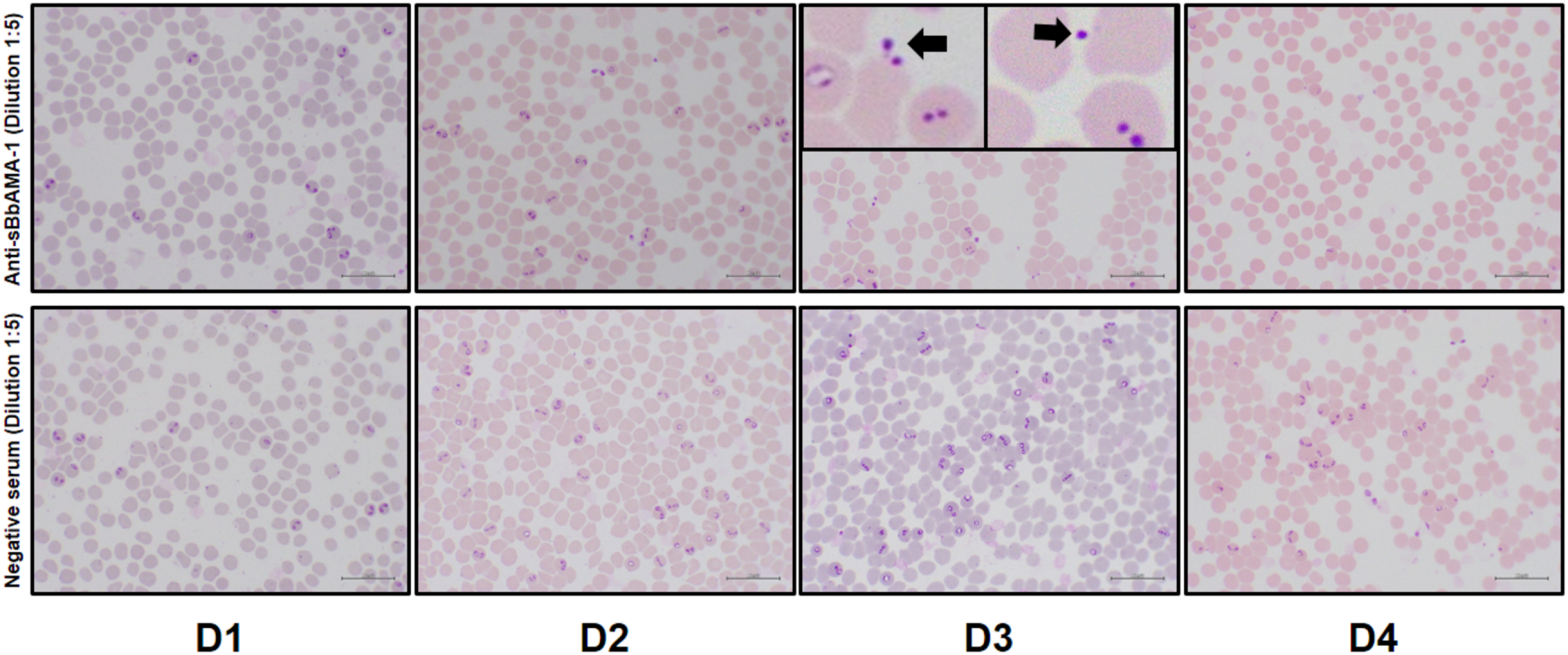
Light microscopic examination of blood smears from *in vitro* cultures treated with rabbit polyclonal anti-sBbAMA-1 antibody. Blood smears prepared with the *B*. *bovis*-infected RBCs from cultures treated with anti-sBbAMA-1serum (upper panel) or rabbit negative serum (lower panel) at 1:5 dilution were observed under a light microscope. D1, D2, D3 and D4 indicate days 1, 2, 3 and 4 post-cultivation. Note the presence of pyknotic extracellular merozoites in the treated cultures indicated by black arrow at day 3. Scale bars = 20 μm.

The microscopic examination of Giemsa-stained blood smears prepared from anti-sBbAMA-1 serum-treated cultures revealed a gradual increase in the number of extracellular merozoites and pyknotic forms when compared to the control cultures (Fig. 5). In particular, these changes were prominent in the cultures that contained anti-sBbAMA-1 serum at a dilution of 1:5.

### *In vitro* invasion-inhibitory effect

**Figure 6:**
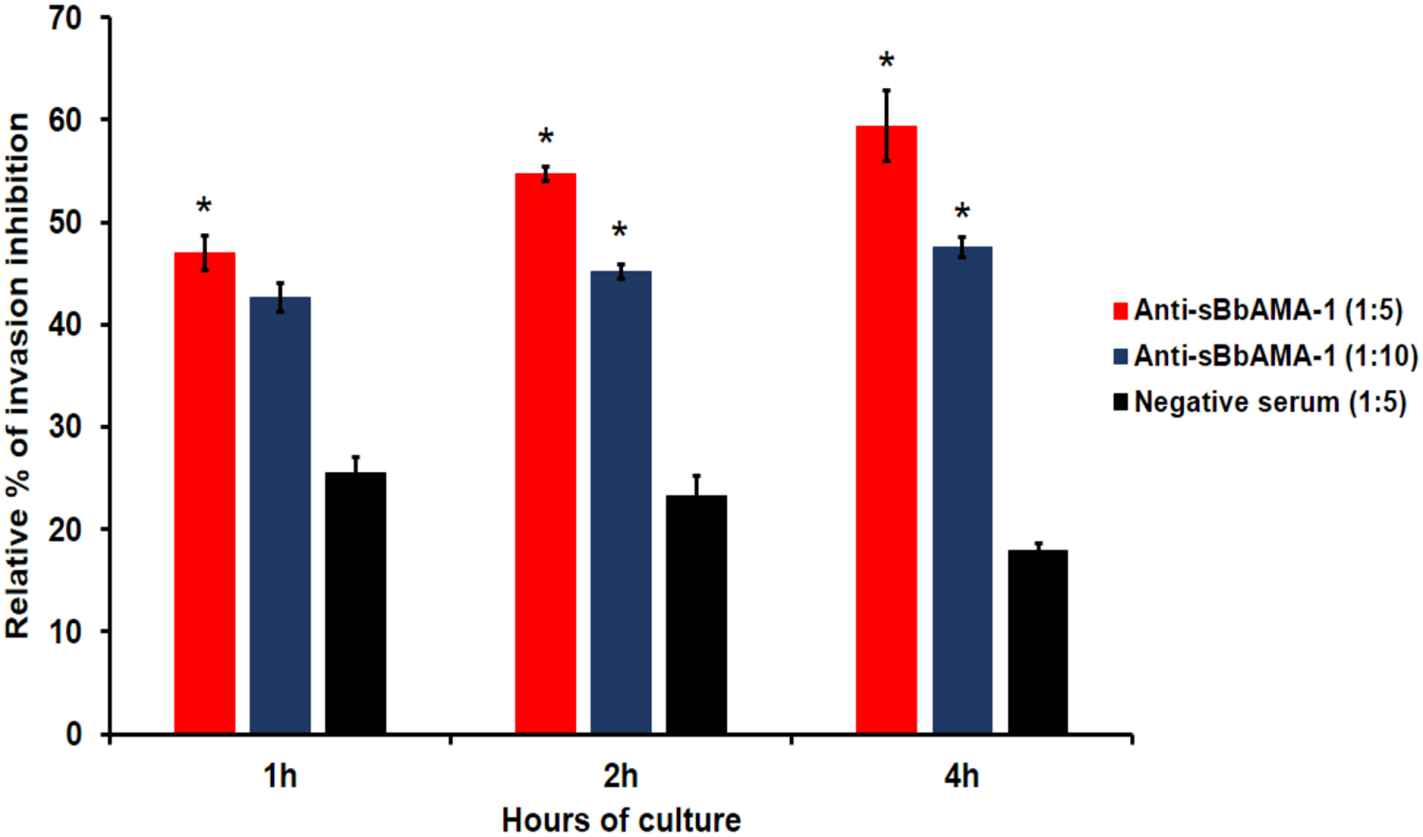
The evaluation of the inhibitory effect of rabbit anti-sBbAMA-1 antibody against *B*. *bovis* merozoite invasion. Purified *B*. *bovis* merozoites were incubated together with fresh bovine RBCs in GIT medium containing rabbit anti-sBbAMA-1 serum at 1:5 and 1:10 dilution or rabbit negative serum at 1:5 dilutions. The percentage of invasion inhibition was calculated based on the parasitemia reduction in anti-sBbAMA-1 antibody-treated cultures compered to GIT-control. Asterisk indicates a significant difference (*p* < 0.05) compared to negative serum control.

To investigate the effects of anti-sBbAMA-1 antibody on the merozoite invasion into RBCs, purified free merozoites isolated from *B. bovis in vitro* cultures were incubated together with fresh bovine RBCs in culture medium containing anti-sBbAMA-1 serum at dilutions of 1:5 and 1:10, while the parasitemia was monitored at 1, 2 and 4 h. The anti-sBbAMA-1 serum had a dilution-dependent effect on the invasion of merozoites into the RBCs. As compared to the GIT-control, we found that the merozoite invasion was significantly inhibited (*p* < 0.05) by approximately 60% in the cultures that contained anti-sBbAMA-1 serum at a dilution of 1:5 dilution after 4 h of incubation (Fig. 6). Furthermore, the degree of invasion-inhibition caused by dilutions of 1:10 and 1:20 at 2 and 4 h was approximately 45%, which was significantly higher when compared with the negative control culture (*p* < 0.05).

## Discussion

Here, we have predicted an epitope from the PAN motif in BbAMA-1 ectodomain I and evaluated its vaccine potential. Interestingly, the sequence alignment of the PAN motif of PfAMA-1 domain I and the corresponding region from BbAMA-1 demonstrated that the BbAMA-1 epitope extends along the conformational epitope region of PfAMA-1 (Bai et al., 2005) and the ligand-binding site of the invasion-inhibitory peptide R1 (Harris et al., 2005). In *P. falciparum*, the PAN motif of PfAMA-1 domain I has been previously identified as the major target of the 1F9 monoclonal antibody (MAb 1F9) (residue 191–247) (Coley et al., 2001, 2006, 2007) and the human anti-AMA1 antibody (humAbAMA1) (residue 194–206) (Maskus et al., 2016), as well as to serve as the partial target of the invasion-inhibitory peptide R1 (Harris et al., 2005; Richard et al., 2010; Vulliez-Le Normand et al., 2012; Lim et al., 2014). These results imply the possibility that the selected BbAMA-1 epitope, which is a target of immune recognition, could induce a protective immunity against *B. bovis* infection.

In the present study, the rabbit antiserum obtained from a rabbit immunized with the synthetic form of the selected epitope reacted positively with *B. bovis* merozoites in IFAT suggesting that the epitope elicits antibodies capable of recognizing the native AMA-1. In Western blot analysis, in agreement with Gaffar et al. (2004), the rabbit anti-sBbAMA-1 antibody reacted specifically with approximately 82-kDa of *B. bovis* protein representing an unprocessed full-length AMA-1. Moreover, consistent with the findings of a previous study (Salama et al., 2013), we detected two additional bands at 69- and 45-kDa which had not been reported by Gaffar et al. (2004). The use of the antibodies that targeted different regions of the BbAMA-1 might be the reason for this discrepancy (Salama et al., 2013). The proteolytic processing of AMA-1 upon merozoite invasion, as demonstrated in several apicomplexan species (Howell et al., 2001; Montero et al., 2009; Moitra et al., 2015; AbouLaila et al., 2019), might explain the presence of multiple bands.

AMA-1 has been extensively studied as an antigen for vaccine development against apicomplexan parasites, especially *P. falciparum*. Several antibodies, such as MAb 1F9 (Coley et al., 2006), humAbAMA1 (Maskus et al., 2016) and the invasion-inhibitory peptide R1 (Harris et al., 2005) raised against the conformational epitopes within the PAN motif of PfAMA-1 domain I, significantly inhibited the invasion of *P. falciparum* into human RBCs. Likewise, in the present study, the rabbit polyclonal antibody raised against the sBbAMA-1 epitope, which is located in the PAN motif of BbAMA-1 domain I and characterized by an α-helix secondary structure, significantly inhibited the growth (50–70% inhibition) and invasion (60% inhibition) of *B. bovis* merozoites. Our results are consistent with those of previous studies (Salama et al., 2013) which found an inhibitory effect of the purified antibody against recombinant BbAMA-1 derived from the central region and ectodomains I and II. The purified IgG at a concentration of 1 mg/ml significantly inhibited the growth and invasion of *B. bovis* merozoites by approximately 40–50% and 60–70%, respectively. Our results are also in agreement with those of a previous study in which a synthetic peptide derived from the N-terminal region of BbAMA-1 domain I significantly inhibited *B. bovis* growth (65% inhibition) (Gaffar et al., 2004). Taken together, these results indicate that the epitope we predicted in the PAN domain I of BbAMA-1 is neutralization-sensitive. The antibodies against sBbAMA-1 may either block some specific functions of AMA-1, be involved in the inhibition of proteolytic processing (Dutta et al., 2003; Gaffar et al., 2004), or block the interaction of AMA1 with its receptor (Montero et al., 2009). This outcome could be a result of merozoite invasion inhibition as evidenced by an increase in the pyknotic forms of the extracellular merozoites in the *in vitro* growth-inhibition assay. Not only is AMA-1 secreted onto the merozoite surface upon invasion, but it is also released at or around the time of merozoite egression (Narum & Thomas, 1994). Therefore, anti-sBbAMA-1 may block merozoite release. This is one possible explanation for the occurrence of pyknotic forms of the intracellular merozoites that reside in infected RBC. The multiple alignment of the BbAMA-1 sequences from various *B. bovis* isolates identified 6 polymorphic sites in the predicted epitope. Therefore, further studies are essential to investigate whether the observed polymorphism results in a degree of strain-specific immunity.

## Conclusions

The present study demonstrated that the rabbit polyclonal antibody against a synthetic peptide derived from the PAN motif of BbAMA-1 domain I is capable of inhibiting the *in vitro* growth and invasion of erythrocytes by *B. bovis* merozoites. Our data suggest that the predicted epitope of the PAN motif of BbAMA-1 domain I may serve as a target antigen for the development of vaccines against bovine babesiosis caused by *B. bovis*.

## Supplemental Information

10.7717/peerj.11765/supp-1 Supplemental Information 1 Original western blots.

10.7717/peerj.11765/supp-2 Supplemental Information 2 Raw data exported from I-TASSER.

10.7717/peerj.11765/supp-3 Supplemental Information 3 Epitope prediction.

10.7717/peerj.11765/supp-4 Supplemental Information 4 Data *in vitro* growth inhibition assay.

10.7717/peerj.11765/supp-5 Supplemental Information 5 Raw data exported from Phyer2.

10.7717/peerj.11765/supp-6 Supplemental Information 6 Data *in vitro* Invasion inhibition assay.

## Additional Information and Declarations

### Data Availability

The following information was supplied regarding data availability: Raw measurements are available in the Supplemental Files.
