# Supplementary figures and images for: Structural and immunological characterization of an epitope within the PAN motif of ectodomain I in Babesia bovis apical membrane antigen 1 for vaccine development
Source: PeerJ. 2021 Jul 16;9:e11765. doi: 10.7717/peerj.11765 (PMC8288113; doi:10.7717/peerj.11765)

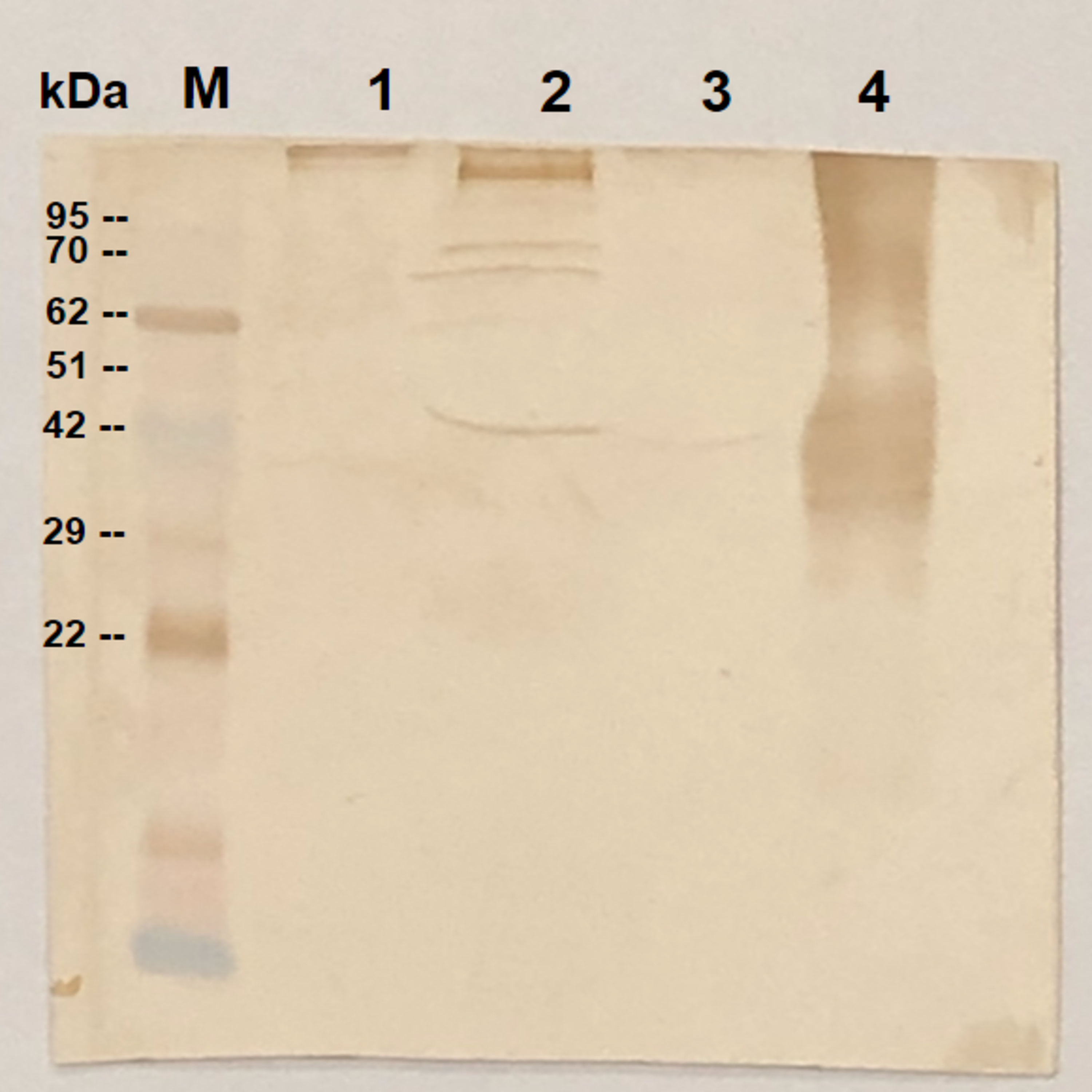

Supplement: Supplemental Information 1 [file peerj-09-11765-s001.png]
